# Identifying predictors of digital competence of educators and their impact on online guidance

**DOI:** 10.1186/s41039-022-00197-9

**Published:** 2022-05-20

**Authors:** Francisco D. Guillén-Gámez, Teresa Linde-Valenzuela, Marta Ramos, María J. Mayorga-Fernandez

**Affiliations:** 1 Department of Didactics and School Organization, Faculty of Education, University of Córdoba, Córdoba, Spain; 2 Department of Didactics and School Organization, Faculty of Education Sciences, University of Malaga (UMA), Malaga, Spain; 3 Department of Developmental and Educational Psychology, School of Education and Tourism of Ávila, University of Salamanca, Salamanca, Spain

**Keywords:** Tutoring action, Teachers, Digital competence, ICT, Research methods, Logistic regression

## Abstract

In the current socio-health situation, new educational challenges have emerged, such as the need to implement a virtual tutorial action. Therefore, this study has three objectives: (1) to investigate the level of digital competence that early childhood and primary school teachers possess to carry out quality online tutorial actions; (2) to analyse whether there are differences in use at both education stages; and (3) to identify which variables significantly affect the development of this competence at each educational stage. For this purpose, an *ex post facto* design was used, based on the survey technique, by means of non-probabilistic purposive sampling. The final sample consisted of a total of 1,069 educators working at the early childhood and primary education stages, from all over Spain. The results showed acceptable digital competence, with higher scores in the primary education stage, which may be due to characteristics of the students and the education stage itself, rather than to teachers’ digital competences. On the other hand, it was found that the virtual tutorial action tasks are significantly influenced in the early childhood education stage by blogs, WhatsApp, Facebook, and number of tutoring hours per month with families, while for the primary stage they are influenced by blogs, WhatsApp, Twitter, ClassDojo, Moodle, tutoring hours, number of tutoring hours per month with families, and sex. Based on these results, there is an obvious need for educational institutions to continue to develop teacher training in relation to the use of resources to carry out adequate tutoring actions and thus increase the diversification in the use of resources.

## Introduction

The pandemic period that began in 2020 has tested teachers’ digital competence, among many other aspects of the education system (Triviño-Cabrera et al., 2021). A considerable number of studies have highlighted the stress suffered by teachers during the first months of enforced confinement (Al-Maroof et al., 2020; Devitt et al., 2020), which continues to affect them (Cerveny, 2021).

This stress on teachers has been caused, in part, by the lack of technology perceived both in schools and in teachers’ and students’ homes (Ramsetty & Adams, 2020). This lack of technology has impacted on the development of tutorial actions implemented with students in early childhood education and primary education (Motaung & Dube, 2020), thus hindering the guidance received, as well as on external educational agents, mainly parents (Donitsa-Schmidt & Ramot, 2020), where teachers’ digital skills have been called into question (Portillo et al., 2020). The problem stems from the fact that, without ensuring the use of information and communication technologies (ICT) in these tasks, tutoring hours may not be able to keep up with the technological advances in most social sectors.

Students are provided with guidance through tutoring which is a mentoring function that every teacher performs, accompanying students in the teaching–learning processes that occur throughout all stages of education (Ripamonti et al., 2018). It is important to point out that guidance in the form of tutoring should not be understood as a one-off process focused solely on problem-solving, but as a sequential and planned process with great pedagogical value (Chafiq & Talbi, 2017). Its functions include the integral formation of the person, responding to the principle of attention to diversity and interculturality (Hernández-Bravo et al., 2017), and, in most cases, having a preventive rather than corrective nature.

In the framework of the European Higher Education Area (EHEA), tutoring is a fundamental teaching function that provides academic, personal, and professional support to students for their personal and professional development (Alegre de la Rosa et al., 2017), as well as having positive effects on students’ academic performance at any educational stage (Guerra-Martin et al., 2017). In this context, Kulik and Fletcher (2016) conducted a meta-analysis of ICT-enabled smart tutoring systems, where 46 of the 50 studies conducted showed a positive effect on student learning.

Since the beginning of the current COVID-19 pandemic, the availability and form of guidance has increasingly been carried out by digital means in order to maintain continuous contact with the education community. Therefore, in addition to the previously mentioned characteristics, in virtual education, a tutor must provide constant feedback (O'Rourke, 2003) and manage virtual platforms (Lentell, 2003) in order to interact in a warmer and more engaging way, as well as to provide the socio-emotional support that students need (De Metz & Bezuidenhout, 2018).

Therefore, to achieve a good orientation and tutoring process through distance processes, a correct digital teaching competence is required. For this, it is necessary to have valid and reliable diagnostic instruments, to establish the starting points of this group. Institutions and research groups are reformulating and developing the concept of digital teaching competence, trying to define and qualify its dimensions. For example, in the European context, the DigCompEdu framework (Digital Competence Framework for Educators) has been developed, with instruments by Ghomi and Redecker (2019). Also with great depth, the TPACK model of Koehler and Mishra (2009) or the PEAT model was developed, which is currently being developed under the framework of the Erasmus + Project «Developing ICT in teacher education (DiCTE, 2019). However, when examining the scientific literature, most of the studies related to tutorial action and student guidance (Espinoza Freire & Ricaldi Echevarría, 2018; Rodríguez López & Llorent Vaquero, 2015) are not related to the use of technology, and consequently, they do not measure the digital skills of teachers to carry out these actions, this being one of the main contributions of this study.

In this digital context, several factors are known to affect digital teaching competencies, such as attitude (Marpa, 2021), age (Lucas et al., 2021), sex (Casillas Martín et al., 2020), years of experience (Ifinedo et al., 2020), self-efficacy (Hammack & Ivey, 2017), flow experience (Calvo-Porral et al., 2017) and area of knowledge (Guillén-Gámez et al., 2021b) can affect their acquisition and development. However, the impact of use of applications such as WhatsApp, Facebook, Instagram, which are used frequently by students (Yunus et al., 2019), has been little analysed within the teaching and learning process, specifically in tutorial action and therefore its impact on the effective delivering of guidance is contested. This may be due to teachers’ lack of digital training regarding the use of said ICT resources. Furthermore, it is necessary to point out that the work of virtual tutorial action does not only take place with the students, but also with the other staff who teach the students, as well as with the families (Wasserman & Zwebner, 2017), offering information, interactivity, support, and motivation towards the new learning environments (Ortega, 2007).

Taking into consideration the digital applications included in previous research, the incidence of demographic and academic variables in the development of teacher training and the contradictory results found in most of these studies, as well as the limited research on online tutorial actions and their impact on guidance, the present study aims to determine the digital competence of early childhood and primary school teachers in terms of their use of ICT resources to carry out online tutorial action tasks. Specifically, the objectives of this study are as follows:

1. To describe the level of digital competence of the teaching staff at both education stages regarding the use of ICT resources to carry out online tutoring activities.
2. To find out whether there are differences in the use of ICT resources between the two education stages.
3. To identify which variables significantly affect the development of teachers’ digital competence at each stage of education.

The present study is relevant for several reasons. First, there are very few studies on this subject (digital resources and tutorial action, especially online); and second, it offers an analysis based on academic and demographic variables that may determine the development of this competence. This will help not only to know what the probability of success is for a teacher to develop an acceptable digital competence, but also to design training strategies that take into account those significant variables, providing framework to improve the effective deliverance of teacher-student guidance.

## Related work and significant variables

Most of the studies observed in the scientific literature on tutorial action have not focused on analysing the impact of digital applications on teacher training. Studies of this type of ICT resources have targeted the teaching–learning process in a general way, with little focus on the framework of tutorial action and even less on the online environment and its impact on the effectiveness of teacher-student guidance.

Among the digital tools analysed in teacher training, the use of blogs has been considered by both early childhood education teachers (Muñoz-Carril et al., 2020) and primary education teachers (Tsetsos & Prentzas, 2021) always highlighting the blog as a learning tool to foster collaborative and critical reflection (Mitchell et al., 2019). For example, a study by Guillén-Gámez et al. (2019) analysed the use of blogs by 134 prospective primary school teachers (English language) according to sex. The results showed that the males obtained a slightly higher score than the females, but there were no significant differences. These results were corroborated in other related studies, such as that carried out by Cakir (2013).

Another tool used by teachers is the social networking platform, Twitter (Nochumson, 2020). For example, Higueras-Rodríguez et al. (2020) analysed, through interviews, the perceptions of 31 primary school teachers, concluding that this social network was ideal for fostering dialogue between teacher and student, favouring both collaborative learning and the development of communication skills. In this sense, Casillas Martín et al. (2020) analysed the knowledge and use made by 332 future early childhood education teachers, finding a high level of competence in the use of this social network. Along the same lines, Barhoumi (2017) analysed the usefulness and ease of use of different digital resources among a sample of 108 educators, finding that Twitter was among the most highly valued resources. Contradictory results were found by Yunus et al. (2019), where the use of this social network in primary education teachers (English) was low.

Regarding instant messaging networks, the use of WhatsApp has been analysed in a number of studies (Casillas Martín et al., 2020; Yunus et al., 2019), identifying a high degree of use of this platform among teaching staff. In relation to tutorial action, Lantarón et al. (2021) assessed the effect of this application in the monitoring and academic tutoring of students, finding a high level of satisfaction in its use, among both students and teaching staff. In the same context, Wasserman and Zwebner (2017) examined communication between teachers and parents through this application, concluding that WhatsApp serves “to circulate an important message very, very fast” (p. 7).

In relation to the use of other platforms, such as Facebook or Moodle, used as learning management systems, Delgado-Garcia et al. (2018) analysed the didactic-pedagogical utilities of these resources, highlighting that, if teachers make appropriate adjustments to these ICT resources, they can be considered new ways of interacting and communicating with students. Along these lines, Casillas Martín et al. (2020) showed that future teachers’ level of mastering the Facebook social network was high, while for Yunus et al. (2019) it was found to be medium.

On the other hand, regardless of the type of digital resource that may be used in the educational process, several studies have highlighted the influence of other variables according to teachers’ digital performance. In regard to the sex variable, some studies have highlighted significant effects on competence levels, generally identifying the males as more competent (Lucas et al., 2021). Other studies have found the opposite (Fernández-Batanero et al., 2019), while others report no differences with respect to specific uses of digital technology (Tondeur et al., 2018) based on sex.

Regarding the variable years of experience and age in relation to level of digital competence, some studies have reported that, with more years of teaching experience, teachers were more likely to have higher digital competence (Benali et al., 2018) while others studies found a negative correlation (Lucas et al., 2021). In a more in-depth analysis, Fernández-Batanero et al. (2019) examined the level of digital competence of 777 in-service teachers concerning the use of ICT resources to assist students with disabilities. The number of years of teaching experience was found to be significant, with a positive relationship with the level of digital competence. Furthermore, the study concluded that there was a gradual increase in teachers’ scores from early childhood education to higher education, with higher scores for the latter. These results were corroborated, in part, by Pegalajar-Palomino (2018), who found significant differences between teachers at these two education stages in two of the four dimensions analysed (didactic implications and professional development). However, the same author in a previous study (Pegalajar-Palomino, 2017) obtained contradictory results regarding the competence level of teachers between these two education stages, where no significant differences were found.

## Method

*Design.* With the purpose of analyzing, comparing and predicting predictors that affect the digital competence of teachers, an *ex post facto* survey design was used. Taking into account the objectives described in depth in the introduction section, the hypotheses are:

- **H1.** The level of digital competence of teachers for tutorial action is low
- **H2.** The level of digital competence of Early Childhood Education teachers for tutorial action is lower than that of Primary Education teachers.
- **H3.** Digital resources and apps have a significant influence on the acquisition of digital teaching competence.

*Sampling* In the latest report from the Ministry of Education, Culture and Sports (MECD, 2020) there were 244,419 teachers. If it were a random sample, with an error of 5%, the sample size would be approximately 384 teachers. Considering the complexity of collecting this type of sampling, it was decided to use purposive sampling, since this technique allows closer access to the subjects, thus with a low production cost. The results of this study should be viewed with caution. This type of design indicates that the sample is not random and, therefore, the results obtained cannot be extrapolated to the general population of Primary and Infant Education. Nevertheless, it will be reflected on the limitations of the study. The sample consisted of a total of 1,069 active early childhood and primary school teachers from 14 communities throughout Spain. Specifically, 21.8% (n = 233) came from the early childhood education stage, with an average age of 41.48 years (± 9.04), while 78.2% (n = 836) came from the primary education stage, with an average age of 43.33 years (± 9.87). Regarding the sex and teaching experience of the teachers, 77.6% (n = 830) were females with an average teaching experience of 16.36 years (± 9.90), while 22.4% (n = 239) were males with an average teaching experience of 16.68 years (± 10.61).

*Instrument.* To measure the digital competence of teachers to carry out online tutorial actions and efficiently offer guidance using ICT, the questionnaire developed by Rufete et al. (2020) and validated by Guillén-Gámez et al. (2021a) in the Spanish population was employed. This instrument consists of 25 items classified into five factors: D1—Tutor functions in relation to students, with five items; D2—Tutor functions in relation to teachers, with six items; D3—Tutor functions in relation to the family, with five items; D4—ICT and transfer, with five items; D5—Use of ICT resources, with four items. A five-point Likert scale was used to measure the items, where the value 1 refers to “no use”, and the value 5 to “frequent use”. Each dimension was focused on the following aspects:

- D1. Digital actions that contribute to the development and enhancement of the basic skills of students, and monitoring-guidance of the educational process.
- D2. Digital skills to work collaboratively, communicate and coordinate with cycle and stage classmates, through ICT resources.
- D3. Digital communication with the parents of the students with the purpose of informing about the educational process of their children, promoting interactivity and providing support.
- D4. Digital skills in the use of technological resources and devices that make up virtual learning environments and web 2.0, in order to fulfill student tutoring functions.
- D5. Digital use of technology resources and devices. Infrastructure of the school in relation to hardware and software which the teacher requires to fulfill his role as tutorial action.

The instrument had satisfactory psychometric properties. This was tested through reliability and validity. The software used for the validation of the instrument as well as for the statistical analyzes were SPSS V.24 and AMOS. V 24. Reliability was measured through different indices: Cronbach's alpha, Spearman-Brown, Guttman, McDonald's Omega, and composite reliability (CR). Table 1 shows the coefficients for each dimension of the instrument. All of them were satisfactory with values higher than 0.7.

**Table 1:** Reliability coefficients

Dimension	*A*	*B*	*C*	*D*	*E*
Cronbach's alpha	.878	.881	.844	.880	.871
Spearman-Brown coefficient	.860	.861	.843	.891	.840
Split-half of Guttman	.826	.858	.816	.891	.840
Omega McDonald	.929	.944	.906	.960	.902
CR	.871	.887	.858	.903	.885

The validity of the instrument was measured through different types. The first of them was through exploratory factor analysis (EFA). The maximum likelihood method with oblique rotations was used. The Kaiser–Meyer–Olkin index was appropriate (KM = 0.949) and the result of the Bartlett Chi-square test was significant (*χ*^2^ = 12,777.009; sig. < 0.05). Therefore, the proposed model explained 59.95% of the true variance in the instrument scores. Specifically, each dimension explained the following proportion of variance: dimension A (7.74%), dimension B (4.95%), dimension C (3.87%), dimension D (38.88%), and dimension E (4.51%). The second type of validity was the convergent which was measured through the average variance extracted (AVE), finding a good model fit with values above 0.50, as recommended by Bagozzi and Yi (1988): D1 (0.585), D2 (0.551), D3 (0.663), D4 (0.567), and D5 (0.616).

Finally, the latent model found in the EFA was tested through confirmatory factor analysis (CFA). The authors followed the recommendations of Bentler (1989): Chi-square ratio over the degrees of freedom (*χ*^2^/g.l; CMIN/DF) taking into account that values below 5 indicate a good fit; the Comparative Fit Index (CFI), the Tucker-Lewis Index (TLI), and Normed Fit Index (NFI), considering values above 0.90 as a good fit; and finally, the Root Mean Square Error of Approximation (RMSEA), where values below 0.07 indicate a good fit of the model. The results in Table 2 show a satisfactory model fit.

**Table 2:** Indicators of goodness of fit model

C.M./df	CFI	TLI	NFI	RMSEA	RMSEA 90% CI
3.551	.923	.914	.901	.068	.063– .073

*Analysis procedure and techniques* Three types of analysis were carried out. The first analysis was descriptive, which determined the mean of each item for each education stage. The second was carried out by testing the normality of the data and its corresponding statistical technique for the two groups. Third, logistic regression analysis was carried out using the stepwise procedure, with the purpose of selecting the best model based on the principle of parsimony (the model with the smallest number of significant variables that explain the highest percentage of an event occurring). To carry out this type of regression, the teachers’ total digital competence score was used (seven-point Likert scale). This score was recoded into a dummy variable with two categories: if the teacher obtained a score below 3, it was recoded with the value 1 (high competence), while if the teacher obtained a score equal to or above 3, it was recoded with the value zero (low competence). The decision of this cut-off value was in relation to the same decisions made by other authors and studies (Cabero‐Almenara et al., 2022; Guillén-Gámez & Ramos, 2021). In addition to the items of the Rufete et al. (2020), the authors asked demographic questions at the beginning of the questionnaire in order to meet the objectives of the study (Table 3).

**Table 3:** Description of variables

Factors	Variable	Type	Measurement scale	Categories
Dependent variable	Global digital competence in tutorial action	Qualitative	Nominal	0: Low 1: High
Personal and academic factors	Sex	Qualitative	Nominal	0: Female 1: Male
	Age	Quantitative	Ratio	
	Teaching experience	Quantitative	Ratio	
	No. of tutoring hours with families per month	Quantitative	Ratio	
	No. of tutoring hours with each student per month	Quantitative	Ratio	
Factors in educational technology	Blogs	Qualitative	Nominal	0: No 1: Yes
	Educational platform of the centre to carry out the tutorial action			
	Instagram			
	WhatsApp			
	TikTok			
	Facebook			
	Twitter			
	Google +			
	ClassDojo			
	Moodle			

## Analysis of the results

This section is divided into three subsections: the first presents the results analysed descriptively by dimension, according to the education stage; the second presents a comparative analysis to identify whether there are significant differences between the dimensions of the instrument according to education stage; and the third analyses the significant variables that affect the overall level of digital competence for teachers at each education stage.

### Level of digital competence by stage

Table 4 shows the teachers’ mean scores for the different items at each education stage. In D1, the item with the highest score was “I provide information to students through digital media (blogs, websites, school educational platform, etc.)”, both for early childhood teachers (*M* = 3.14) and primary teachers (*M* = 3.74), which showed a higher score regarding the first one. On the other hand, the item, “I propose digital strategies to students to identify erroneous information or fake news” showed very low scores, being lower for early childhood education school teachers (*M* = 1.45) than for primary school teachers (*M* = 2.44). In D2, the item with the highest score was “I coordinate with the rest of the teaching team of the class group through different digital media…” with high and similar means for both early childhood teachers (*M* = 4.14) and primary teachers (*M* = 4.17). The item with the lowest score, although having a medium value on the five-point Likert scale, was “I propose online collaborative environments to work with the teaching team regarding my tutoring activity”, with a lower score for early childhood teachers (*M* = 2.87) than for primary school teachers (*M* = 3.13). In D3, the item with the highest score was, “I make presentations in digital format for group meetings with families”, where the score was slightly lower for early childhood school teachers (*M* = 3.20) compared to primary school teachers (*M* = 3.51). It can be seen that the remainder of the items in this dimension scored average values on the five-point Likert scale, where early childhood school teachers had a slightly lower score than primary school teachers for all items. In D4, the item with the highest score was “I produce digital content related to tutoring in a safe and responsible way”, with a slightly lower score for early childhood teachers (*M* = 3.54) compared to primary school teachers (*M* = 3.61). Finally, it can be seen that the use of ICT resources was similar among the teaching staff at both stages, as the scores were similar for all items. These scores can be interpreted as medium–high levels with respect to the five-point Likert scale.

**Table 4:** Descriptive analysis according to education stage

D1- Tutor’s functions in relation to the student body	Early childhood education			Primary education		
	M ± SD	*A*	*K*	M ± SD	*A*	*K*
-I provide information to students through digital media (blogs, websites, educational platform)	3.14 ± 1.45	− 0.19	− 1.29	3.74 ± 1.13	− 0.57	− 0.63
-I provide learners with strategies for communicating safely online	2.19 ± 1.39	0.79	− 0.75	3.12 ± 1.25	− 0.18	− 0.95
-I teach students how to solve accessibility and e-inclusion problems	1.76 ± 1.15	1.49	1.25	3.11 ± 1.24	− 0.13	− 0.93
-I propose digital strategies to students to identify misinformation or fake news	1.45 ± 0.92	2.32	1.95	2.44 ± 1.23	0.43	− 0.82
-I set students tasks with the technologies that involve collaboration between them	2.16 ± 1.34	0.83	− 0.64	2.97 ± 1.31	0.03	− 1.08
D2- Duties of the tutor in relation to the teaching staff						
-I coordinate with the rest of the teaching team of the class group through different digital media (videoconferences, chats, WhatsApp groups)	4.14 ± 1.09	− 1.09	0.25	4.17 ± 0.96	− 0.99	0.30
-I carry out classroom planning collaboratively using editable online documents	3.72 ± 1.25	− 0.66	− 0.59	3.59 ± 1.30	− 0.56	− 0.79
-Telematic tools are available to develop common lines of action with the other tutors within the school’s tutorial action plan	3.41 ± 1.28	− 0.29	− 0.96	3.48 ± 1.22	− 0.43	− 0.75
-I use digital resources to establish common guidelines for tutorial action with the rest of the tutors	3.33 ± 1.32	− 0.22	− 1.07	3.29 ± 1.22	− 0.23	− 0.87
-The teaching team has defined strategies to solve the difficulties of accessibility and inclusion of students	3.28 ± 1.33	− 0.31	− 1.00	3.43 ± 1.13	− 0.32	− 0.57
-I propose online collaborative environments to work with the teaching team on aspects of my tutoring	2.87 ± 1.36	0.14	− 1.16	3.13 ± 1.26	− 0.11	− 0.99
D3- The tutor’s role with the family						
-Development of digital educational projects involving the educational community (families, teachers and students) in relation to the education centre	2.64 ± 1.35	0.27	− 1.09	2.71 ± 1.30	0.22	− 1.02
-I make digital presentations for group meetings with families	3.20 ± 1.44	− 0.23	− 1.25	3.52 ± 1.36	− 0.44	− 1.06
-I offer guidance to families on the possible use of technologies at home in accordance with their child’s educational needs	3.01 ± 1.34	− 0.01	− 1.16	3.31 ± 1.15	− 0.20	− 0.78
-I provide families with strategies to solve problems of accessibility and digital inclusion and to communicate through technology for educational purposes for their children	2.95 ± 1.37	− 0.05	− 1.20	3.25 ± 1.17	− 0.25	− 0.77
-I advise families on the responsible use of ICT at home by their children	2.97 ± 1.38	− 0.06	− 1.20	3.21 ± 1.22	− 0.19	− 0.92
D4- TIC and transference						
-I have received ongoing training on technologies as a means of using them in tutorial action	2.87 ± 1.26	0.14	− 0.97	3.03 ± 1.27	− 0.03	− 1.04
-I actively develop my digital competence related to mentoring	3.18 ± 1.23	− 0.16	− 0.96	3.44 ± 1.16	− 0.28	− 0.80
-I address accessibility and technology inclusion as part of mentoring	2.93 ± 1.24	0.03	− 0.96	3.28 ± 1.15	− 0.15	− 0.80
-I carefully plan the use of ICT to ensure their added value	3.13 ± 1.28	− 0.09	− 0.97	3.39 ± 1.14	− 0.32	− 0.70
-I produce digital content related to mentoring in a safe and responsible manner	3.54 ± 1.26	− 0.50	− 0.69	3.61 ± 1.16	− 0.49	− 0.61
D5- Use of ITC resources						
-The school has resources to carry out tutorial action through technology	3.53 ± 1.38	− 0.55	− 0.93	3.64 ± 1.30	− 0.62	0.− 0.74
-I use ICT easily to co-ordinate with the teaching team during the current online teaching period	3.91 ± 1.17	− 0.85	− 0.10	4.00 ± 1.03	− 0.80	− 0.11
-I use ICT easily to interact with learners during the current period of online teaching	3.55 ± 1.35	− 0.50	− 0.95	3.90 ± 1.08	− 0.70	− 0.33
-I use ICT easily to co-ordinate with families, during the current period of online teaching	3.93 ± 1.15	− 0.85	− 0.19	3.93 ± 1.06	− 0.72	− 0.29

*M = average; *SD* standard deviation; *A* asymmetry; *K* Kustosis

**Fig. 1:**
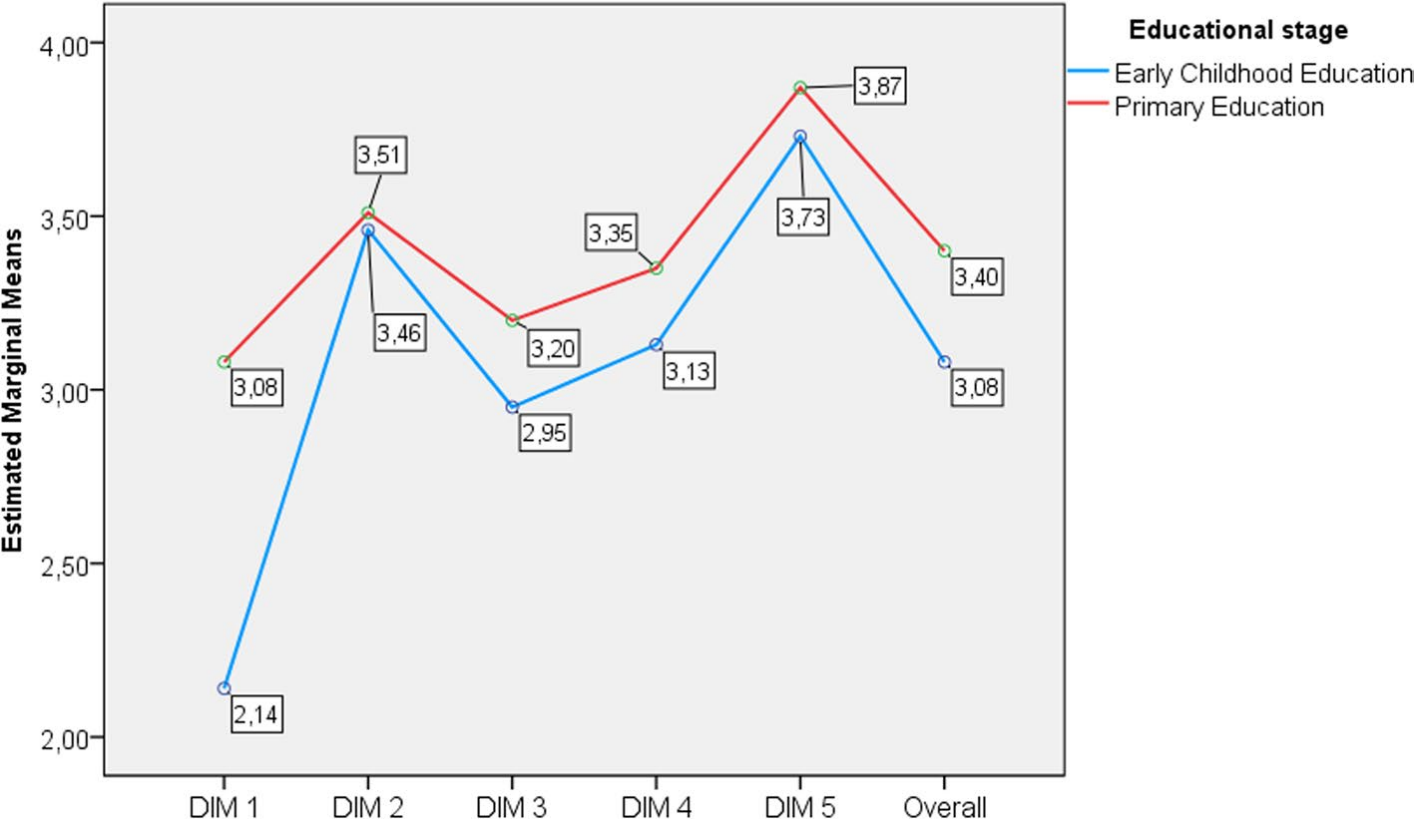
Overall score and average score for each dimension of the instrument for each education stage

In order to compare the differences found in Table 1 between the teachers at each education stages, the scores for the different items were grouped by dimension and given an overall value. Figure 1 shows the teachers’ level of digital competence in online tutoring for each of the dimensions of the instrument, as well as their overall level of digital competence in regard to the instrument, for each of the samples (early childhood education teachers and primary education teachers). It can be seen that there was a big difference in D1, where early childhood education teachers scored lower (*M* = 2.14) than primary teachers (*M* = 3.08). In the rest of the dimensions, it can be observed that there was a difference of approximately half a point between the two stages (for D3, D4, D5, and overall), with these scores always being higher in primary school teachers, with the exception of D2, where the scores were similar.

### Differences in digital competence by stage

In order to check whether the differences found in the scores presented in Fig. 1 with respect to the teachers’ educational stage were significant, a statistical analysis of these scores was carried out (Table 5). The Mann–Whitney U test found that there were significant differences in the level of competence between teachers at both stages in D1 with a large effect size (*d* = 0.828), D3 with a small effect size (*d* = 0.186), D4 with a small effect size (*d* = 0.178), and finally, overall digital competence, with a small to medium effect size (*d* = 0.319).

**Table 5:** Comparison of each dimension of the instrument between the two education stages

Dimensions	Kolmogorov–Smirnov		Mann–Whitney			Effect size (d)
	KS	*p*	*U*	*Z*	*p*	
D1- Duties of the tutor in relation to the student body	.075	.001	48,116.500	− 12.519	.001*	0.828
D2- Duties of the tutor in relation to the teaching staff	.068	.001	98,365.500	− 0.675	.500	–
D3- The tutor’s role with the family	.073	.001	88,351.500	− 3.036	.002*	0.186
D4- ICT and transfer	.072	.001	88,919.000	− 2.903	.004*	0.178
D5- Use of ICT resources	.130	.001	94,647.000	− 1.561	.119	–
Global digital competence	.043	.001	79,334.000	− 5.153	.000*	0.319

*Significance level at 0.05

As significant differences were found in some dimensions as well as in the overall competence of the instrument between teachers at both education stages, we proceeded to carry out the logistic regression technique separately for each educational stage, thus identifying which variables specifically affected the development of overall digital competence in online tutorial action tasks at each education stage.

### Predictors of digital competence by stage

The prediction of the significant variables affecting the level of teachers’ digital competence was carried out by analysing the total score of the instrument through the technique based on multiple logistic regression. The assumptions that allowed the logistic regression technique to be carried out for both types of sample were tested. In the case of early childhood education teachers, the monotonicity assumption was tested through the Hosmer and Lemeshow test which showed correct results (*χ*^2^ = 7.898; gl = 8; sig. = 0.443). The multicollinearity assumption was also tested and no collinearity problems were found between the selected variables, as the tolerance values were found to be greater than 0.6 (Chan, 2004). With regard to primary education teachers, the monotonicity assumption was satisfactory (*χ*^2^ = 6.002; gl = 8; sig. = 0.657), while the multicollinearity assumption test found no tolerance values lower than 0.6.

The omnibus test estimated that the proposed model was significant, both for early childhood teachers (*χ*^2^ = 51.454; gl = 15; *p* < 0.05) and primary teachers (*χ*^2^ = 74.917; gl = 15; *p* < 0.05). The goodness of fit of the model was tested using the Nagelkerke regression coefficients (early childhood = 0.338; primary = 0.131) and Cox and Snell regression coefficients (early childhood = 0.189; primary = 0.087), which showed that the model explains between approximately 8.7% and 13.1% for primary teachers and between 18.9% and 33.8% for early childhood teachers. It was also found to be able to correctly predict 89% of the cases for early childhood teachers and 77.3% for primary teachers, meaning the models were acceptable.

Table 6 shows the significant predictors of having an acceptable level of digital competence in online tutoring, both for early childhood teachers and primary teachers. For example, it can be seen that, for early childhood teachers, the variables that significantly influenced the development of their digital competence were: blogs, WhatsApp, Facebook, and the number of tutorials with families per month; while for primary teachers they were: blogs, WhatsApp, Twitter, ClassDojo, Moodle, and the number of tutorials with families per month.

**Table 6:** Predictors of the likelihood of achieving an acceptable level of digital competence

	*β*		Sig		Exp(B)	
	Early childhood education	Primary education	Early childhood education	Primary education	Early childhood education	Primary education
Blogs	1.169	0.603	0.019	0.001	3.218	1.828
Centre platform	0.202	0.087	0.759	0.751	1.224	1.091
Instagram	0.733	− 0.713	0.280	0.124	2.080	0.490
WhatsApp	1.001	− 0.393	0.038	0.045	2.720	0.675
TikTok	− 0.333	0.244	0.746	0.593	0.717	1.277
Facebook	1.436	0.339	0.009	0.271	4.205	1.403
Twitter	− 1.514	1.137	0.175	0.001	0.220	3.118
Google	− 0.098	0.175	0.842	0.371	0.907	1.192
ClassDojo	0.331	0.418	0.487	0.021	1.393	1.519
Moodle	0.880	0.499	0.069	0.006	2.410	1.647
Tutoring activities with families	0.455	0.133	0.013	0.037	1.576	1.143
Tutoring activities with students	− 0.025	0.038	0.727	0.132	0.976	1.039
Age	0.034	− 0.031	0.487	0.146	1.034	0.970
Teaching experience	− 0.076	0.010	0.109	0.623	0.927	1.010
Sex	− 1.437	0.565	0.109	0.003	0.238	1.759
Constant	− 8.498	− 3.608	0.000	0.000	0.000	0.027

Significance level at 0.05

As such, it is possible to calculate the probability a teacher has of acquiring an acceptable digital competence depending on whether or not he/she uses the variables that were found to be significant in the model, by means of the following formula:

$$p (Y=1)=\frac{1}{1+{e}^{-({\beta }_{0}+{\beta }_{1}{\mathrm{x}}_{1}+\cdots +{\beta }_{k}{\mathrm{x}}_{k})}}$$

For example, for an early childhood school teacher who uses blogs and Facebook to communicate news to families, uses a WhatsApp group to communicate information about meetings or collaborative projects and, in addition, usually has two tutorial activities per month for each family, such an educator would have a probability of achieving an acceptable digital competence in ICT resources to carry out tutorial action of 41.48%; while for a female primary school teacher who uses the variables found to be significant (blogs, WhatsApp, Twitter, ClassDojo, Moodle, tutorial activities with families) on a daily basis, she would have a probability of achieving an acceptable digital competence of 37.98%.

## Discussion

This study has analysed the digital competence of early childhood education and primary education teachers in terms of their use of ICT resources to deliver guidance in alternative forms of tutoring.

With regard to the first objective, both early childhood and primary school teachers demonstrated acceptable digital competence in the use of ICT resources to carry out online tutoring activities, in all the different dimensions. In this sense, it was rejecting the hypothesis no. 1.The level of digital competence shown by teachers at both education stages seems logical, bearing in mind that they have studied educational sciences as part of the social sciences area. Guillén-Gámez et al. (2021b) showed that professionals in this area have a high level of digital competence. Moreover, this result corroborates the finding that all tutors provide support, even online, in the teaching–learning process in any education stage (Ripamonti et al., 2018). Moreover, it corroborates the idea that tutorial action is sequential and planned, as found by Chafiq and Talbi (2017).

In relation to the second objective, early childhood and primary school teachers demonstrated different use of ICT resources to carry out online tutoring activities which in turn effected their ability to offer effective guidance. Although teachers at both education stages showed an acceptable level of competence, it was significantly lower in the case of early childhood teachers. These general results would confirm hypothesis nº2.In line with previous studies (Fernández-Batanero et al., 2019), there appeared to be a gradual increase in the use of these resources when moving from early childhood education to higher education stages, such as primary education. An evolutionary interpretation might explain this: during the six years that children are enrolled in primary education (from 6–12 years of age), they experience numerous cognitive, biosocial, and psychosocial changes that influence and condition their teaching–learning process (Berger, 2007) compared to the three years (3–6 years of age) that the child spends in the second cycle of early childhood education. Without underestimating the important changes that take place in this period, logically, in the first case, the tutor must give more support and guidance and carry out a longer and more demanding accompaniment of students, family, and teachers.

More specifically, significant differences with a small effect size were found in D3 “Tutor functions in relation to the family” and D4 “ICT and transfer”, whereas in D1 “Tutor’s role in relation to students” where differences were marked by a large effect size. This is not surprising given the nature of these items. It is logical that an early childhood teacher does not offer students digital strategies to identify misinformation or fake news while a primary school teacher does, for two reasons. One relates to the development of reading skills: While early childhood education students are immersed in the acquisition of encoding/decoding mechanisms, primary education students—with the exception of those with Specific Learning Difficulties associated with reading—focus on more complex processes, such as text comprehension. In other words, students at both education stages are at different points (attainment, alphabetic, or orthographic) in the process of becoming expert readers (Frith, 1985). The second reason may be that students in early childhood education have less maturity and autonomy to use ICT resources, which is why it is essential that teachers at this stage lay the foundations for students’ subsequent development (Martínez Redondo et al., 2014), and why the use of these resources represent an approximation to them. The third reason could be the informal daily contact that exists in Early Childhood Education (De Vega, 2009). These face-to-face contacts at specific times such as the arrival or departure of school would allow teachers and parents to exchange information about the minor without the need to use ICT for it.

Only in D2 (Functions of the tutor in relation to the teaching staff) and D5 (Use of ICT resources) was no difference in use found between teachers at the two education stages. Thus, it seems that both early childhood and primary school teachers use ICT resources such as videoconferencing, e-mail or WhatsApp to coordinate with the rest of the teaching staff, and also use these tools easily in online tutoring tasks. It is logical to assume that there are no differences when establishing coordination between peers and that these resources are again easily used, taking into account the acceptable level of digital competence of the teaching staff shown in this study, as well as in other previous studies (Guillén-Gámez et al., 2021b).

In short, it is likely that the differences found between teachers at both education stages are not due to a lack of knowledge about the use of ICT resources, but rather to the nature of the education stage and students themselves conditioning their use in online tutoring tasks. In fact, no significant differences were found in the use with teaching staff (D2) and the use of ICT resources (D5), while the greatest differences were found in relation to the use with students (D1) and with families (D3).

With regard to the third objective, the digital competence of early childhood and primary school teachers in tutorial action tasks seems to be significantly conditioned by the use of blogs, WhatsApp, and the number of tutorials per month with families. These data would partially corroborate hypothesis 3 by finding significant predictors in some resources and not in all; as well as in those results obtained in previous research. For example, in relation to WhatsApp, there is a significant positive feeling regarding its use in tasks related to the monitoring and academic tutoring of students (Lantarón et al., 2021), as well as a considerable percentage of use of this tool to facilitate communication of academic (Minhas et al., 2016). These results demonstrated that, also in this specific area -online tutorial action-, the extensive use of WhatsApp, previously shown by Casillas Martín et al. (2020) and Yunus et al. (2019) in early childhood education and primary education teachers, respectively. As for blogging, similar results were obtained in both early childhood education (Muñoz-Carril et al., 2020) and primary education (Tsetsos & Prentzas, 2021) contexts.

It seems logical that these three applications, among the evaluated ones, are the most influential ICT resources in the digital competence of teachers in both educational stages, taking into account the nature of the tutorial action, regardless of the modality. For example, WhatsApp allows constant (O'Rourke, 2003) and rapid feedback (Wasserman & Zwebner, 2017), facilitating communication between parents, teachers, and tutors, whereas blogs are a learning tool that allows for richer, collaborative, more flexible and critical reflection to be encouraged (Mitchell et al., 2019) which provides the opportunity to have tutoring hours with families.

Although resources have been identified that influence the digital competence of teachers at both education stages, this study has shown there are specific ones at each stage that promote good participation in the virtual platforms necessary for tutorial action (Lentell, 2003). For example, Facebook seems to affect only the digital competence of early childhood teachers, while Twitter, ClassDojo, and Moodle affect the digital competence of primary teachers. In the specific case of Facebook, these results corroborate the findings of previous studies in which a high level of mastery of this social network was obtained by early childhood education teachers (Casillas Martín et al., 2020) compared to a lower level of mastery by primary education teachers (Yunus et al., 2019). In relation to Twitter, Higueras-Rodríguez et al. (2020) consider it to be an ideal social network for fostering dialogue between teacher and student, favouring both collaborative learning and the development of communication skills. Indeed, Casillas Martín et al. (2020) identified a high competence in this social network in early childhood school teachers, although the results obtained does not seem to predict such information, at least for online tutorial action. Furthermore, these results contradict those reported by Yunus et al. (2019), who reported a low use of this social network in primary school teachers. As for Moodle, it seems to be an influential tool in the development of competence; this is logical considering that it is a new way of communicating and interacting with students (Delgado-Garcia et al., 2018).

## Conclusions

The unexpected emergence of Covid-19 has presented challenges around the world, and the education system has been no exception. Spanish schools were paralysed by the pandemic, forcing them to migrate all educational activity to the online mode. This forced the implementation of strategies to improve the teaching–learning process (Du Plessis & Mestry, 2019), and especially the processes of offering guidance, using ICT.

As such, tutors took on the role of providing personalised support to students, their families, and other teachers (Wasserman & Zwebner, 2017). Tutorial action, therefore, contributes to providing a global education aimed at fostering the comprehensive development of students in intellectual, affective, personal, and social dimensions, which has positive effects on the academic performance of students at any stage of education (Guerra-Martin et al., 2017), even when ICT resources are used (Kulik & Fletcher, 2016). The challenge ahead is to strengthen and sustain this achievement. Therefore, higher education institutions, within the framework of the European Higher Education Area, must not let their guard down but must continue to train and consolidate these digital competences. Only in this way will future tutors be prepared to design, select, and implement strategies that respond to the specific needs of the different members of the education community using ICT resources.

The use of ICT resources such as blogs, WhatsApp, and tutoring hours celebrated in a monthly basis with the family in the case of early childhood and primary school teachers, plus Facebook for the former, and Twitter, ClassDojo, and Moodle for the latter, has implications for education practice. They should be considered as tools to carry out not only activities related to the content of disciplines, but also other activities such as tutorial actions, ensuring a teaching–learning process that is adapted to the twenty-first century (Kale, 2018).

Although studies of these characteristics allow us to advance in the knowledge of specific variables linked to the digital competence of Spanish early childhood and primary school teachers that would allow us to improve online tutorial action, they also have certain limitations. On the one hand, a convenience sample was used; thus, the sample does not represent the full range of Spanish teachers at both education stages. On the other hand, social biases may have influenced the responses to the self-report questions. Finally, the parents could have influenced the use of digital resources and Apps for the tutorial action of their children, since these resources cannot be used by the students in the sample (mainly Early Childhood Education students).

Future research should expand on studies in this area in three ways: first, by using other samples in order to generalise the results to the whole population under study; second, evaluating the role of parents in their children's use of resources and aps for online tutoring; and, third by exploring the level of satisfaction of the students, families, and other teachers involved in online tutoring with the tutoring that is received. If the reflections on digital skills are carried out from a triangular prism, that is, from the different agents involved in the teaching–learning process

## Data Availability

Not applicable.
